# Total Blood Exosomes in Breast Cancer: Potential Role in Crucial Steps of Tumorigenesis

**DOI:** 10.3390/ijms21197341

**Published:** 2020-10-05

**Authors:** Maria Konoshenko, Georgy Sagaradze, Evgeniya Orlova, Tatiana Shtam, Ksenia Proskura, Roman Kamyshinsky, Natalia Yunusova, Antonina Alexandrova, Anastasia Efimenko, Svetlana Tamkovich

**Affiliations:** 1 Institute of Chemical Biology and Fundamental Medicine, Siberian Branch of Russian Academy of Sciences, 630090 Novosibirsk, Russia; msol@ngs.ru (M.K.); ksen-84@list.ru (K.P.); 2 Medical Research and Education Center, Lomonosov Moscow State University, 119991 Moscow, Russia; georgysagaradze@gmail.com (G.S.); efimenkoan@gmail.com (A.E.); 3 N.N. Blokhin Cancer Research Center” of the Ministry of Health of the Russian Federation, 115478 Moscow, Russia; evg19111976@yandex.ru (E.O.); tonya_alex@yahoo.com (A.A.); 4 Petersburg Nuclear Physics Institute named by B.P. Konstantinov of National Research Center “Kurchatov Institute”, 188300 Gatchina, Russia; shtam_ta@pnpi.nrcki.ru; 5 National Research Center “Kurchatov Institute”, 123182 Moscow, Russia; kamyshinsky.roman@gmail.com; 6 Novosibirsk Regional Clinical Oncological Dispensary, 630108 Novosibirsk, Russia; 7 Moscow Institute of Physics and Technology, 141700 Dolgoprudny, Moscow region, Russia; 8 Shubnikov Institute of Crystallography of Federal Scientific Research Centre, “Crystallography and Photonics” of Russian Academy of Sciences, 119333 Moscow, Russia; 9 Cancer Research Institute, Tomsk National Research Medical Center, Russian Academy of Science, 634050 Tomsk, Russia; bochkarevanv@oncology.tomsk.ru; 10 Department of Biochemistry and Molecular Biology, Faculty of Medicine and Biology, Siberian State Medical University, 634050 Tomsk, Russia; 11 Department of Molecular Biology and Biotechnology, Faculty of Natural Sciences, Novosibirsk State University, 630090 Novosibirsk, Russia

**Keywords:** exosomes, migration, angiogenesis, cryo-electron microscopy, microRNAs, breast cancer

## Abstract

Exosomes are crucial players in cell-to-cell communication and are involved in tumorigenesis. There are two fractions of blood circulating exosomes: free and cell-surface-associated. Here, we compared the effect of total blood exosomes (contain plasma exosomes and blood cell-surface-associated exosomes) and plasma exosomes from breast cancer patients (BCPs, *n* = 43) and healthy females (HFs, *n* = 35) on crucial steps of tumor progression. Exosomes were isolated by ultrafiltration, followed by ultracentrifugation, and characterized by cryo-electron microscopy (cryo-EM), nanoparticle tracking analysis, and flow cytometry. Cryo-EM revealed a wider spectrum of exosome morphology with lipid bilayers and vesicular internal structures in the HF total blood in comparison with plasma. No differences in the morphology of both exosomes fractions were detected in BCP blood. The plasma exosomes and total blood exosomes of BCPs had different expression levels of tumor-associated miR-92a and miR-25-3p, induced angiogenesis and epithelial-to-mesenchymal transition (EMT), and increased the number of migrating pseudo-normal breast cells and the total migration path length of cancer cells. The multidirectional effects of HF total blood exosomes on tumor dissemination were revealed; they suppress the angiogenesis and total migration path length of MCF10A, but stimulate EMT and increase the number of migrating MCF10A and the total path length of SKBR3 cells. In addition, HF plasma exosomes enhance the metastasis-promoting properties of SKBR3 cells and stimulate angiogenesis. Both cell-free and blood cell-surface-associated exosomes are involved in the crucial stages of carcinogenesis: the initiation of EMT and the stimulation of proliferation, cell migration, and angiogenesis. Thus, for the estimation of the diagnostic/prognostic significance of circulating exosomes in the blood of cancer patients more correctly, the total blood exosomes, which consist of plasma exosomes and blood cell-surface-associated exosomes should be used.

## 1. Introduction

Exosomes are small endosome-derived vesicles (30–120 nm) that are secreted by multiple cell types into the extracellular space [1]. The size of these vesicles allows them to penetrate from various tissues into blood and then back, interacting with target cells or tissues. Exosomes carry multiple receptors and ligands on their surface that are responsible for the biodistribution and binding of vesicles to target cells [2,3]. This allows the exosomes to transfer different types of RNA and functionally active proteins, providing cell-to-cell communication [4,5]. 

Increasing attention is paid to the pathological role of exosomes produced by cancer cells in tumorigenesis. These vesicles are able to carry molecular messages from transformed to healthy cells or to other cells in the tumor, or they may signal in an autocrine manner, causing changes in the recipient cell’s behavior and microenvironment alterations [6]. Notably, exosomes may deliver signals, inducing proliferation [7], migration [5,8], invasion [9], angiogenesis [10], and epithelial-to-mesenchymal transition (EMT) [11]. Since the cargo of exosomes depends on the cell of origin, tumor-derived exosomes in blood circulation represent a promising biomarker for monitoring tumor progression and minimal residual disease [6,12,13,14]. However, the detection, isolation. and profiling of cancer-specific exosomes from blood plasma is still challenging, since the share of such vesicles does not exceed 1–2% [15,16].

It should be noted that blood circulating exosomes have been reported to contain distinguishable fractions of cell-free and cell-surface-associated vesicles [17,18,19]. It is known that circulating blood exosomes contact with blood cell plasma membranes. It can be assumed that after such interactions, some exosomes remain cell-surface-associated for a while, without vesicle membrane fusion/internalization with a cell membrane. Indeed, TEM and flow cytometry revealed no morphological differences between cell-free and blood cell-surface-associated circulating vesicles [17,18]. However, it has been shown that the proteins and miRNAs from blood cell-surface-associated exosomes represent valuable sources of biomarkers for breast cancer diagnostics [17,18]. For example, the level of miR-103, which stimulates tumor metastasis and angiogenesis by targeting Dicer and Ago1, respectively [20,21], in blood cell-surface-associated exosomes from breast cancer patients (BCPs) is significantly higher compared to that of healthy volunteers [17]. Moreover, the level of miR-195, which directly downregulates WNT3A [22], in blood cell-surface-associated exosomes is lower in cancer patients than in healthy persons. The combination of miR-103 and miR-195 in the fraction of exosomes associated with red blood cells enabled researchers to distinguish luminal BCPs at early stages with a sensitivity of 71% and a specificity of 89%, whereas the same analysis in the fraction of cell-free exosomes enabled researchers to distinguish cancer individuals only with a 50% sensitivity and 67% specificity [17]. Thus, we suppose that for the estimation of the diagnostic/prognostic significance of blood circulating exosomes of cancer patients, it is more correct to use total blood exosomes containing both plasma exosomes and blood cell-surface-associated exosomes.

In the present study, we isolated exosomes from the plasma and total blood of healthy female donors (HFs) and BCPs to directly compare their possible roles in crucial steps of tumor progression by elucidating their effects on the proliferation, epithelial-to-mesenchymal transition (EMT), cell migration, and stimulation of angiogenesis. 

## 2. Results

### 2.1. Characterization of Exosomes Isolated from Plasma and Total Blood

**Figure 1:**
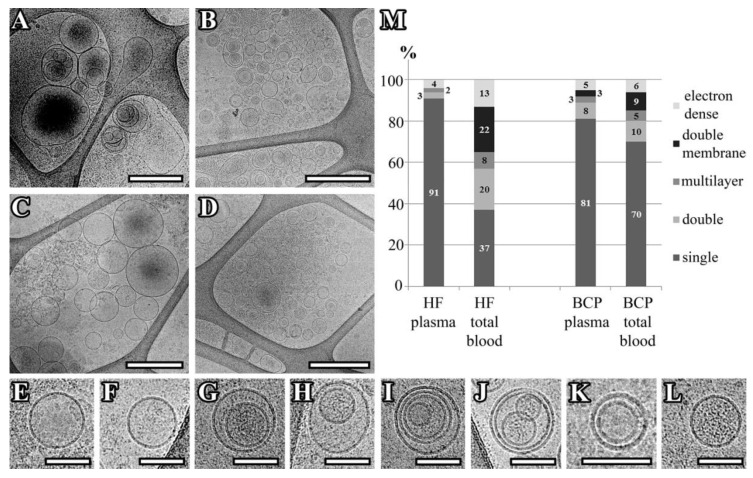
Representation of exosomes with various morphologies. A–L: Cryo-EM images of exosomes isolated from pooled samples of the plasma of HFs (**A**), Total blood of HFs (**B**), plasma of BCPs (**C**), total blood of BCPs (**D**), a single vesicle (**E**,**F**), double vesicles (**G**,**H**), multilayer vesicles (**I**,**J**), double-membrane vesicle (**I**–**K**), vesicles with an electron-dense cargo in lumen (**G**,**L**). Scale bars are 500 nm for micrographs (**A**–**D**) and 100 nm for micrographs (**E**–**L**). **M**: Percentages of single, double, double-membrane, multilayer, and vesicles with an electron-dense cargo visualized in the vesicle samples of pooled plasma and total blood from HFs and BCPs.

The morphology of the extracellular vesicles (EVs) isolated from the plasma and total blood of HFs and BCPs was examined by cryo-electron microscopy (cryo-EM). In total, the images of 367 vesicles and 303 vesicles from the plasma and total blood of HFs (Figure 1a,b) and the images of 154 vesicles and 301 vesicles from the plasma and total blood of BCPs (Figure 1c,d) were analyzed. More than 90% of EVs were identified as exosome-like vesicles due to the clear presence of a lipid bilayer/membrane. Most of the exosome-like vesicles could be classified as single (Figure 1e,f), double (Figure 1g,h), double-membrane (Figure 1i–k), multilayer (Figure 1i,j), and electron-dense cargo-contained vesicles (Figure 1g,l). Most of the vesicles were intact and had a round or slightly elongated shape. Single (Figure 1a–f), double (Figure 1a–d,g,h), and multilayer exosome-like vesicles (Figure 1a–d,i,j) were visualized in all samples of isolated vesicles from the pooled plasma and pooled total blood of HFs and BCPs. Exosome-like vesicles were assigned into a multilayer category when two or more vesicles were contained inside a larger one. It was found that the multilayer vesicles were larger than the single ones. Besides double vesicles, vesicles with two membranes bilayers were found which fit tightly to each other around the whole circumference (Figure 1J). Protein aggregates outside EVs were also visualized.

It should be noted that single vesicles were predominantly represented in the samples of HF plasma, while in the total blood vesicles their content decreased (from 91% to 37%) with an increase in double vesicle (from 3% to 20%) and double-membrane vesicle (from 0% to 22%) fractions (Figure 1M). The morphology of the vesicles isolated from the plasma and total blood of BCPs was comparable with that of the prevailing single vesicles (81% and 70%, respectively) (Figure 1M). 

**Figure 2:**
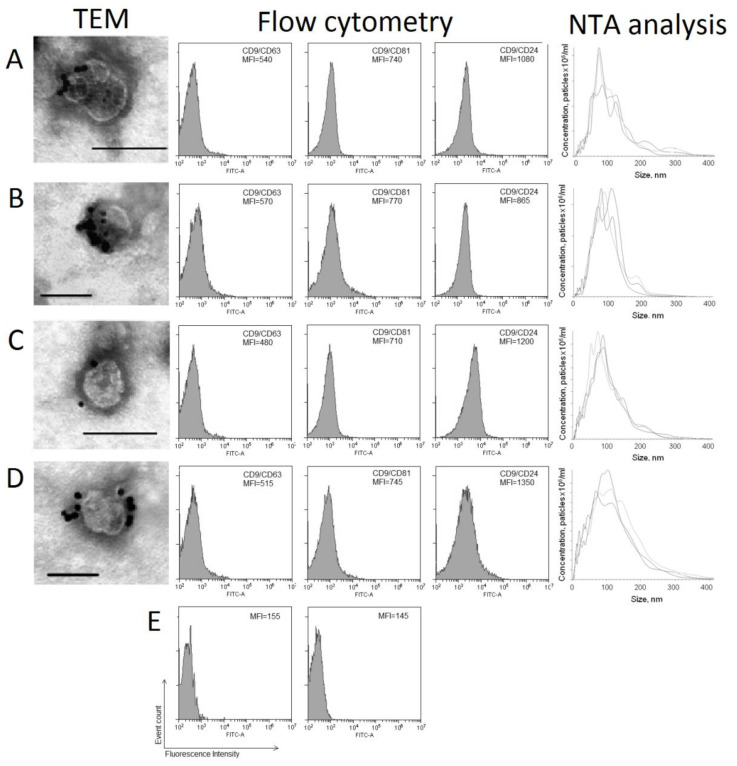
Exosomes characterization by immunogold labeling-TEM, flow cytometry, and NTA analysis. For immunogold labeling, exosomes were incubated with monoclonal antibodies to CD9, with subsequent detection by the conjugate of protein A and gold nanoparticles. Scale bars correspond to 100 nm. Negative staining is shown by phosphotungstic acid. For flow cytometry, the mean MFIs are shown. Blood plasma of HFs (**A**), total blood of HFs (**B**), blood plasma of BCPs (**C**), total blood of BCPs (**D**), isotype control and negative control (latex beads labeled anti-CD9 with anti-CD81 FITC antibody) (**E**).

Nanoparticle tracking analysis (NTA) showed that the EVs from HF plasma had a mean size of 114 nm, with a mode of 77 nm and an SD of 63 nm (Figure 2a). The total blood EVs had a mean size of 101 nm, with a mode of 94 nm and an SD of 42 nm (Figure 2b). Similarly, the EVs from BCP plasma had a mean size of 105 nm, with a mode of 84 nm and an SD of 59 nm (Figure 2c). The total blood EVs had a mean size of 111 nm, with a mode of 97 nm and an SD of 50 nm (Figure 2d).

The isolated exosome-like vesicles were characterized for the presence of exosomal markers using immune-gold staining and flow cytometry (Figure 2). A subpopulation of CD9/CD24-positive EVs predominated in both the plasma and total blood of HFs and BCPs. There were no significant differences in the median fluorescence intensity (MFI) of the CD9/CD24-positive, CD9/CD63-positive, and CD9/CD81-positive EV population between the plasma and total blood of HFs and BCPs.

Collectively, our data demonstrate that EVs isolated from the plasma and total blood of both HFs and BCPs share the same characteristics as exosomes.

### 2.2. Exosomes from Plasma and Total Blood of BCPs Promote Tube Formation by Endothelial Cells

**Figure 3:**
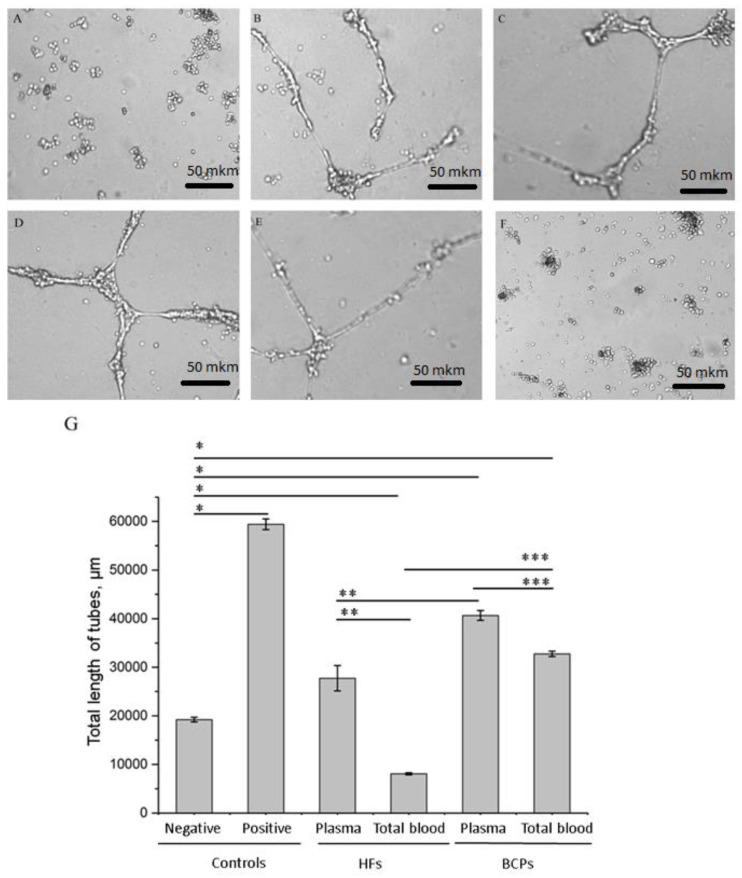
The effects of plasma and total blood exosomes on tube formation in HUVECs. (**A**–**F**): Representative images showing tube formation in HUVECs treated with PBS (negative control) (**A**), exosomes from the plasma (B) and total blood (**C**) of BCPs, 10% FBS (positive control) (**D**), exosomes from the plasma (**E**) and total blood (**F**) of HFs. Scale bar is 50 mkm. (**G**) Quantitative analysis of the tube formation assay. The values for the total tube length were measured (mean ± SEM, * *p* < 0.05 vs. negative control, ** *p* < 0.05 vs. exosomes from plasma of HFs, *** *p* < 0.05 vs. exosomes from total blood of BCPs).

Angiogenesis is a term that describes the formation of new blood vessels from a pre-existing vasculature [23]. The effects of exosomes from plasma and total blood from HFs and BCPs on angiogenesis were evaluated using in vitro tube formation assays. We observed a more extensive network of capillary-like structures formed by human umbilical vein endothelial cells (HUVECs) on Matrigel in the presence of exosomes from plasma and the total blood of BCPs as compared to the negative control (*p* = 0.0027 and *p* = 0.0030, respectively); moreover, the BCP plasma exosomes had a more pronounced effect compared to the BCP total blood exosomes and HF plasma exosomes (*p* = 0.0206 and *p* = 0.0439, respectively) (Figure 3). 

Furthermore, incubation with exosomes from the total blood of HFs decreased the tube formation capability in comparison with the negative control, exosomes from HF plasma, or BCP total blood (*p* = 0.0024, *p* = 0.0173 and *p* = 0.0006, respectively) (Figure 3).

### 2.3. Exosomes from Plasma and Total Blood Influence Tumor Cell Migration

**Figure 4:**
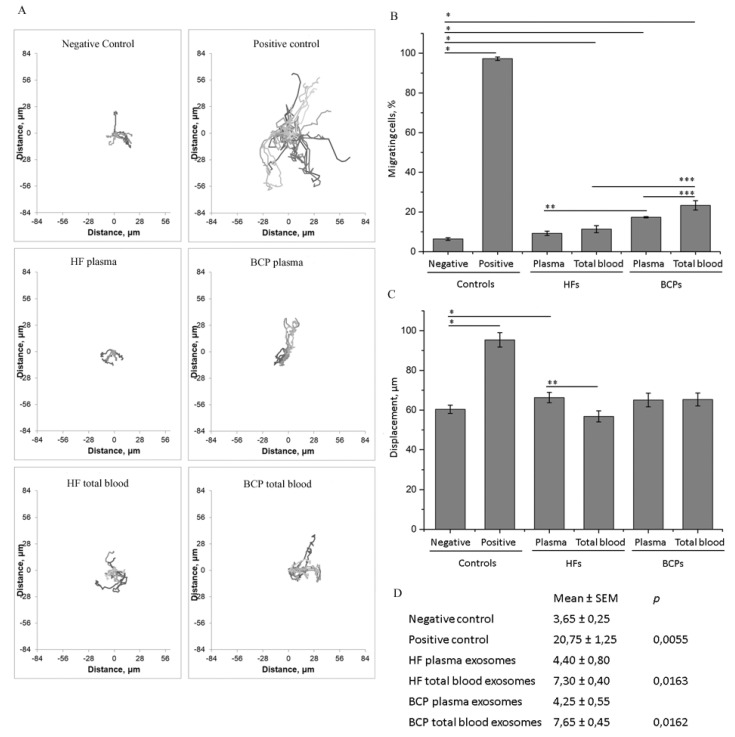
The effects of plasma and total blood exosomes on MCF10A cell migration and proliferation. Results of three independent experiments are presented as mean ± SEM, * *p* < 0.05 vs. negative control, ** *p* < 0.05 vs. exosomes from plasma of HFs, *** *p* < 0.05 vs. exosomes from total blood of BCPs. Trajectory plots of single-cell migration experiments of MCF10A in the presence or absence of exosomes (**A**), the proportion of motile cells (**B**), displacement over 15 h (**C**), and mitotic activity (percentage of mitosis events during 15 h of observation) (**D**).

To evaluate the ability of exosomes from plasma and total blood from HFs and BCPs to modulate tumor cell migration, we used the non-malignant breast cell line MCF10A and the breast cancer cell line SKBR3. Epithelial MCF10A cells were almost immobile under serum-free and epidermal growth factor (EFG)-free conditions (negative control). The addition of serum and/or EGF to cells (positive control) significantly stimulated their motility (Figure 4a). 

Thus, the number of migrated cells (Figure 4b) and the migration path (Figure 4c) increased significantly (*p* = 0.0062 and *p* < 0.0001, respectively). The addition of exosomes from the total blood of HFs or from the plasma and total blood of BCPs resulted in a significant increase in the motile cell number compared to the negative control (*p* = 0.0369, *p* = 0.0253, and *p* = 0.0253, respectively) (Figure 4a,b). Nonetheless, the total path length of MCF10A cells was found to be reduced after the addition of HF total blood exosomes in comparison with exosomes from the plasma of HFs (*p* = 0.0219) (Figure 4c).

**Figure 5:**
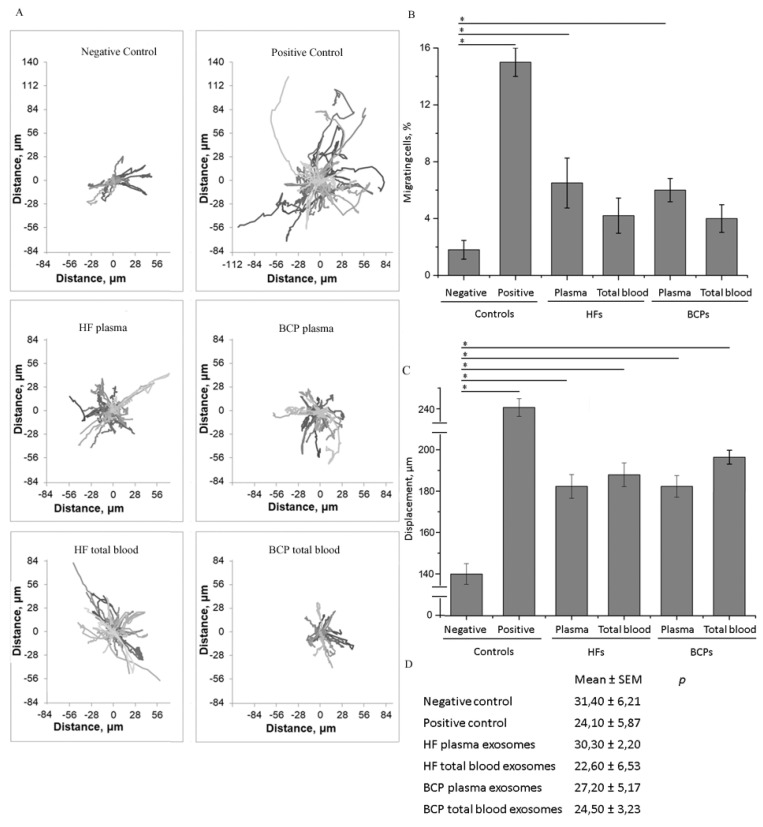
The effects of plasma and total blood exosomes on SKBR3 cell migration and proliferation. Results of three independent experiments are presented as mean ± SEM, * *p* < 0.05 vs. negative control. Trajectory plots of single-cell migration experiments on SKBR3 in the presence or absence of exosomes (**A**), the proportion of motile cells (**B**), displacement over 15 h (**C**), and mitotic activity (percentage of mitosis events during 15 h of observation) (**D**).

Breast cancer SKBR3 cells were mainly represented by single cells or cells combined in small groups. In the presence of 10% FCS without exosomes (positive control), many motile cells were observed (Figure 5a). The wash out of FCS (negative control) led to a significant weakening of cell migration, suggesting that FCS had the greatest impact on the SKBR3 cell motility. Particularly, the number of motile cells decreased significantly (*p* = 0.00001) (Figure 5b). The addition of exosomes from plasma of HFs and BCPs to the negative control cells increased the amount of motile tumor cells by 3.6 and 3.3 times (*p* = 0.0373 and *p* = 0.0106, respectively); the effects of the addition of total blood exosomes from both HFs and BCPs were less pronounced (Figure 5a,b).

It should be noted that motile SKBR3 cells were observed even in dense culture and inside groups which moved actively even without additional treatments; the migration path of motile cells was affected significantly by the addition of FCS or exosomes in comparison to the negative control (*p* < 0.0001 for FCS and BCP exosomes, *p* = 0.0002 for HF plasma exosomes, *p* = 0.0010 for HF total blood exosomes) (Figure 5c). 

### 2.4. Influence of Exosomes on Cell Proliferation Intensity

In the case of non-tumor MCF10A cells, where proliferation was highly sensitive to the presence of serum and/or growth factors (*p* = 0.0055), the addition of total blood exosomes both from HFs and BCPs led to a statistically significant increase in the mitotic event number (*p* = 0.0163 and *p* = 0.0162, respectively) (Figure 4d). On the contrary, the proliferation of cancer SKBR3 cells, which effectively proliferated without serum, was not affected by the addition of any type of exosomes (Figure 5d).

### 2.5. The Morphology of E-Cadherin and β-Catenin-Based Adherence Junctions (AJs) in Tumor Cells after Treatment by Exosomes

**Figure 6:**
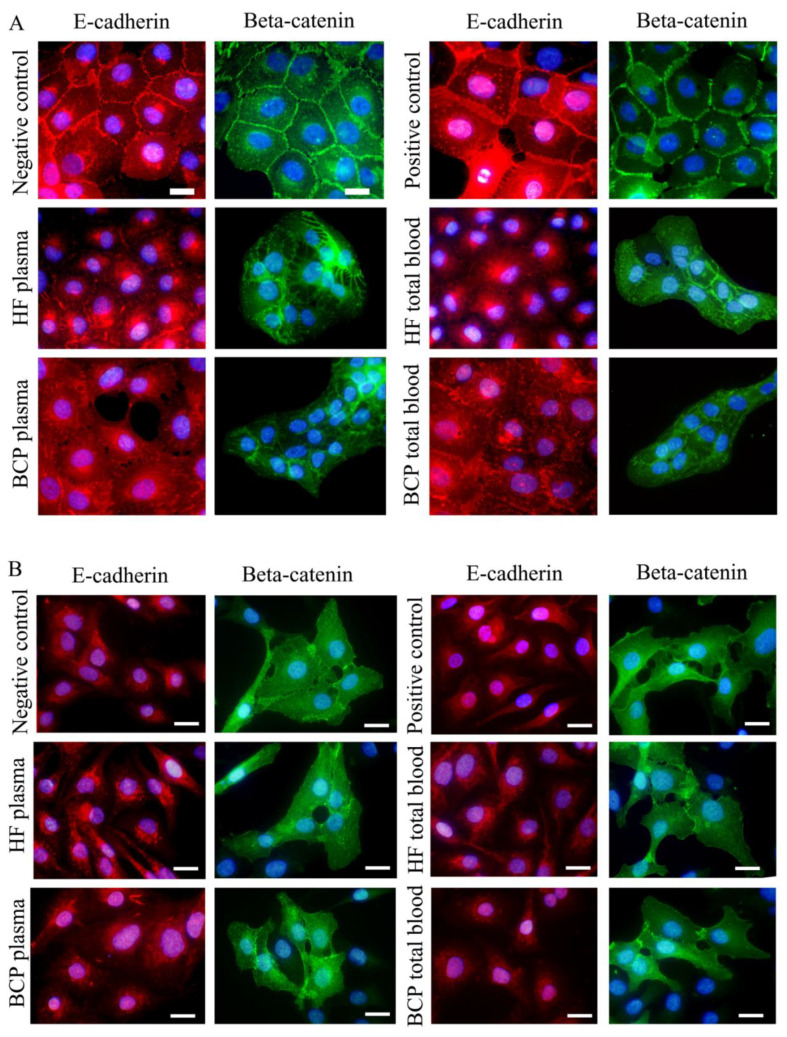
The influence of exosomes on the morphology of cell-cell adhesions of cells by immunofluorescent staining of E-cadherin (red color) and β-catenin (green color). Cell nuclei stained with DAPI (blue). Experimental conditions are described near the pictures. Bar is 20 µm. MCF10A cells (**A**), SKBR-3 cells (**B**).

To assess the influence of blood-derived exosomes on the ability of tumor cells to form Adherence Junctions (AJs), we analyzed the expression of E-cadherin and β-catenin, as key proteins within intercellular contacts, in MCF10A and SKBR3 cells by immunofluorescent staining. MCF10A epithelial cells formed small islands on the glass surface in the absence of horse serum and EGF (negative control), and demonstrated well-pronounced E-cadherin and β-catenin-based AJs, which were organized as adhesion belts along the cell–cell boundaries (Figure 6a). The addition of horse serum and EGF (positive control) led to a significant alteration of AJ morphology. The E-cadherin staining at the boundaries between cells became more diffuse; the cell edges overlapped and at the areas of cell overlapping both E-cadherin and β-catenin adhesions were organized as clouds of small dots (Figure 6a). Similar types of cell–cell adhesions were shown earlier during the trans-epithelial migration of transformed rat epithelial cells [24]. The addition of any fraction of blood exosomes (plasma or total) from HFs and BCPs to cells led to a significant disruption of E-cadherin-based AJs. Only the residual adhesion structures remained visible, and they were represented by randomly distributed dots or small radial structures (Figure 6a). The β-catenin-positive AJs also reorganized significantly. In cells treated with exosomes, β-catenin-based AJs were better pronounced than E-cadherin-based AJs and represented non-regular radial structures at the overlapping areas of neighbor cells. The disruption of AJs was associated with the appearance of small gaps between cells (Figure 6a). 

We also studied the influence of exosomes on intracellular adhesions in SKBR3 cells. These cells were not shown to form E-cadherin-based AJs with or without the presence of any studied type of exosome (Figure 6b), even when they contacted in groups or islands. Thus, we used staining for β-catenin to reveal cell–cell adhesions in contacting cells. In the negative control, these adhesions were represented by short tangential lines, radial adhesions, or drop-like adhesions at the overlapped edges of cells. SKBR3 cells did not form pronounced cell–cell adhesions—there were several small gaps between cells (Figure 6b). 

The addition of FCS to SKBR3 cells led to an increase in cell separation, and even contacting cells did not form β-catenin-based adhesions. The addition of total blood exosomes either from HFs or BCPs to SKBR3 cells did not significantly change the number and morphology of the β-catenin-based cell–cell adhesions. 

Thus, we did not observe any influence of total blood exosomes on the morphology of the intercellular adhesions of highly motile SKBR3 cancer cells which did not organize E-cadherin-based AJs. However, the total blood exosomes both from HFs and BCPs altered the intercellular adhesions of epithelial MCF10A cells and thus stimulated the first steps of EMT.

### 2.6. Over-Expression of miR-25-3p and miR-92a in Exosomes from BCP Blood

**Figure 7:**
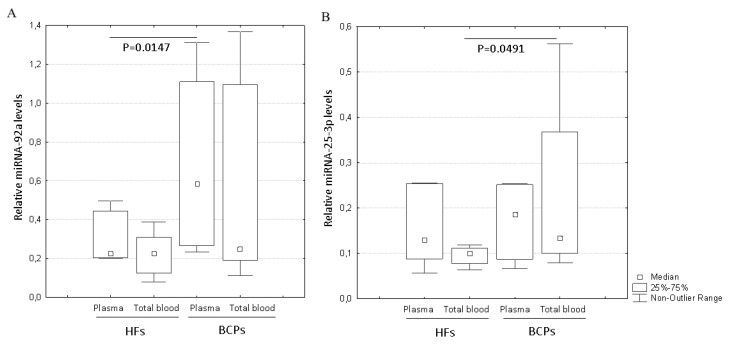
Quantification of exosomal miRNAs from the plasma and total blood of HFs and BCPs. Tukey box plots of miRNAs. The statistically significant *p*-values are indicated. miR-92a (**A**), miR-25-3p (**B**).

A number of previous studies have indicated that miRNAs, being one of the main functional components of exosome cargo, may play a crucial role in cell-to-cell communication and eventually regulate the biological functions of recipient cells. It is known that miR-92a and miR-25-3p are up-regulated in cell lines with enhanced EMT, cell motility, and angiogenesis [25,26,27,28,29,30]. Since the miR-16-5p expression was stable and reproducible, it was chosen as an endogenous control to normalize the miRNA expression [30,31,32]. For microRNAs, qRT-PCR assays with a working range of 24–39 threshold cycles (Ct) of PCR were used. Non-template controls produced no signal or were at least seven cycles away from the minimum detectable amount of specific template. All the reported data were obtained using RNA samples that produced Ct values within the working range of the systems. Spike-in control (cel-miR-39) was detected in all the samples at 25 ± 1 Ct. Data on the miRNA relative expression in exosomes from the total blood of HFs and BCPs are presented in Figure 7.

The miR-92a relative expression in plasma exosomes of BCPs was significantly higher compared to that of the HFs (*p* = 0.0147) (Figure 7a). As about total blood exosomes, only the median exosomal miR-25-3p level was significantly higher in BCPs than in healthy volunteers (*p* = 0.0491) (Figure 7b).

Since the samples of exosomes from total blood contain plasma exosomes and blood cell-surface-associated exosomes, we conclude that some of the cancer-derived exosomes may circulate in plasma, and some of these exosomes are associated with the blood cell surface. Moreover, the primary data demonstrated that cell-associated exosomes play an important role in the development of tumor progression, transferring miRNAs involved in the development of angiogenesis, cell motility, and EMT.

## 3. Discussion

The importance of exosomes in governing various physiological and pathological conditions is no longer in doubt. Exosomes may transfer signals to induce EMT, cell migration and invasion, angiogenesis, and metastasis processes [5,8,9,10,11]. It should be noted that the majority of studies were performed with exosomes from cell culture. At the same time, tumor cell lines differ by molecular signatures such as p53 mutation, loss of heterozygosity, nucleotide mutation, different gene expression level, neuroendocrine differentiation, and cytogenetic markers. Due to this heterogeneity the effects induced by exosomes may differ between cell lines. There is a complex mixture of circulating vesicles of different origin in blood, including a large number of exosomes of hematopoietic and endothelial origin, as well as tumor exosomes [33,34]. Most studies regarding the involvement of blood exosomes in carcinogenesis focus on the fraction of plasma exosomes. However, another fraction of exosomes—blood cell-surface-associated exosomes—is similarly noteworthy [17,35]. Obviously, exosomal transport is provided not only by the liquid blood fraction but also by blood cells. However, the effects of cell-bound exosomes on normal and tumor cells have not yet been elucidated.

Our previously published data indicated that total blood exosomes morphologically resemble plasma exosomes, and both pools of exosomes were positive for CD24, CD63, CD81, and CD9 markers [35]. However, cryo-EM has established a wide spectrum of exosome morphology with lipid bilayers and vesicular internal structures. To the best of our knowledge, only two studies have examined several novel morphological EVs subcategories from human blood plasma by cryo-EM [36,37], and none in human total blood. We revealed a variety of exosomes (single, double, double with two membrane bilayers, and multilayer vesicles), which was in accordance with previously obtained data [36,37]. Double and multilayered vesicles containing electron dense material were also visualized. These types of EVs were described earlier in ejaculate and cerebrospinal fluid [38,39]. For the first time, we have found that single exosomes predominate in the plasma of HFs and in the plasma and total blood of BCPs, while their proportion in the total blood of HFs is reduced by almost 2.5 times. Since total blood exosomes contain two fractions (cell-free exosomes and blood cell-surface-associated exosomes), the revealed phenomenon indicates that exosomes of various morphologies predominate on the surface of blood cells. Therefore, we have suggested that subpopulations of exosomes with different and specific functions do exist, and blood cells act as their transporters. Moreover, these subpopulations might be redistributed in cancer.

Earlier, we found that the proportion of blood cell-associated exosomes isolated from blood of BCPs is decreased if compared to HFs [17,35]. Since most exosomes in the blood of cancer patients are of non-tumor origins, the reason for this decrease remains unclear. The detected phenomenon may be associated with a decreased amount or affinity of receptors to exosomes on the blood cell surface membrane. As a result, the increased concentration of exosomes in plasma of cancer patients allows exosomes to more efficiently provide cell-to-cell communication through horizontal transfer of bioactive molecules affecting tumor progression. 

It was shown that EVs isolated from the triple-negative breast cancer cells HCC1806 were capable to induce the proliferation and drug resistance of the non-tumorigenic MCF10A breast cells. MiRNA profiling in the recipient cells suggested that these phenotypes could be mediated by changes in the expression of miRNAs associated with proliferation, apoptosis, invasion, and migration [40]. Moreover, earlier it was found that exosomes from ovarian cancer cells SKOV-3 and OVCAR-3 promoted mesenchymal stem cell and endothelial cell migration in a time- and concentration-dependent manner [41].

Not only the effects of tumor-derived exosomes on normal cells, but also the effects of exosomes secreted by normal tissue cells on cancer cells are of importance for disease pathogenesis. For example, plasma exosomes of different origins affect circulating tumor cells and exosomes secreted by stromal cells affect tumor cells. Indeed, it was shown that exosomes from healthy donors’ plasma have potency in stimulating the metastasis-promoting properties of breast cancer cells [5].

In the present study, we examined the effects of plasma exosomes and total blood exosomes from HF and BCP blood on the key steps of tumor progression: proliferation of tumor cells, EMT, cell migration, and the stimulation of angiogenesis. In vitro analysis revealed that exosomes from plasma of BCPs stimulated morphological alteration of cell-cell adhesions typical for earlier stages of EMT of MCF10A and enhanced angiogenesis better than all other types of exosomes. Moreover, we have shown that the BCP plasma exosomes significantly increased the number of motile pseudo-normal and the number of motile and total path of tumor cells. The observed biological effects of BCP plasma exosomes could be due to the increased miR-92a expression in these vesicles, which we have demonstrated in the current study for the first time. This assumption is supported by previous studies which have shown that the increased level of miR-92a correlates with increased angiogenesis and cell migration activity [25,26].

Additionally, we revealed that exosomes from the total blood of BCPs stimulate the EMT of MCF10A cells and capillary-like structures formation. Moreover, the treatment of non-tumor MCF10A cells by exosomes from the total blood of BCPs increased the number of motile and proliferating cells significantly. Similar processing of SKBR3 cells enhanced the total path length of these cells. We also demonstrated the significantly elevated miR-25-3p level in the total blood exosomes from BCPs in comparison to HFs. This is consistent with known data about the association between the high miR-25-3p expression and both increased angiogenesis and the cell migration activity [27,28,29]. Thus, the plasma exosomes and total exosomes from the blood of BCPs caused similar (but not identical) biological effects and were characterized by different expressions of tumor-associated miRNAs. 

Since plasma exosomes constitute only a part of total blood exosomes, it is more correct to estimate the altered expression of microRNAs associated with the processes critical for the development of breast cancer in total blood exosomes. Considering their potential role in tumorigenesis, total blood exosomes may serve as promising targets for therapy—for example, to prevent neoangiogenesis and migration of cancer cells.

On the contrary, exosomes from HF total blood suppressed capillary-like tube formation by HUVEC and the path length of breast pseudo-normal cells in comparison to exosomes from HF plasma, but significantly increased the motile MCF10A cell numbers and the number of mitosis events. It was an unexpected finding that all the blood exosomes (from the plasma and from total blood) of HFs stimulated the EMT of MCF10A cells and the total path of SKBR3 cells. The ability of plasma-circulating exosomes to potentiate tumor malignant properties was observed in some previous studies [42,43,44]. The similar results were revealed for exosomes from healthy donors as well as for MDA-MB-231 and MCF-7 breast cancer cells [5]; plasma exosomes stimulated the adhesive properties, two-dimensional random migration, and transwell invasion of breast cancer cells in vitro as well as their in vivo metastatic dissemination in a dose-dependent manner.

Thus, exosomes in the blood of healthy women behave as the two-faced Janus: they reduce the aggressive behavior by normal cells and have potency in stimulating the metastasis-promoting properties of breast cancer cells. Since most exosomes in the blood of cancer patients are of non-tumor origin, it is likely that, without attracting attention to them, exosomes from normal cells play a significant role in tumor dissemination.

## 4. Materials and methods

### 4.1. Ethics Statement

The study protocol was approved by the Ethics Committee of the Institute of Chemical Biology and Fundamental Medicine (the protocol N10 from 30.05.2018). Human samples were obtained according to the principles expressed in the Declaration of Helsinki. Written informed consent was obtained from every female. Blood samples from HFs (*n* = 35, age 22–78 years, median age 40) were obtained from Novosibirsk Central Clinical Hospital; blood samples from previously untreated BCPs (*n* = 43, age 32–81 years, median age 60) were obtained from Novosibirsk Regional Oncology Dispensary (Table 1). 

### 4.2. Exosome Isolation

Venous blood (9 mL) was collected in K_3_EDTA spray-coated vacutainers (Improvacuter, China, cat. no. 694091210), immediately mixed using a rotary mixer, placed at +4 °C, and processed within an hour after blood taking. 

The blood sample was divided into two equal parts. One part was used for the isolation of plasma exosomes, and the second was used for the isolation of total blood exosomes. To isolate the plasma exosomes and total blood exosomes by ultrafiltration and differential ultracentrifugation, the previously described protocol was used [35]. 

To study the impact of exosomes on cell migration and angiogenesis, half of the exosome samples from different individuals were mixed, to generate two samples (plasma exosomes and total blood exosomes) from HFs, and two samples (plasma exosomes and total blood exosomes) from BCPs. Individual and mixed samples were frozen in liquid nitrogen and stored in aliquots at −80 °C until required. The aliquots were thawed once before use.

### 4.3. Exosomes Characterization

The morphology of the isolated EVs was assessed by cryo-EM, as described previously [38]. The initial volumes of plasma or blood for the study of EV using the cryo-EM were 10 mL.

The size and concentration of the isolated EVs were evaluated by NTA using the NanoSight^®^ LM10 (Malvern Instruments, UK) analyzer, equipped with a blue laser (45 mW at 488 nm) and a C11440-5B camera (Hamamatsu Photonics K.K., Japan), at several dilutions according to the manufacturer’s instructions. Each sample was measured in triplicate, with a camera setting of 15, an acquisition time of 60 s, and a detection threshold setting of 5. At least 200 completed tracks were analyzed per video. NTA analytical software version 2.3 was used for data analysis and capture.

The immunocytochemical identification of exosomes from plasma and total blood with monoclonal antibodies to tetraspanin CD9 was performed as described previously [45]. Quantitative analysis of the exosomal tetraspanines on the surface of the isolated EVs was carried out using flow cytometry, as described previously [46]. Flow cytometry was performed on the Cytoflex (Becman Coulter, USA), using the CytExpert 2.0 Software. The MFI of the stained exosomes was analyzed and compared to the isotype control (BD bioscience, USA).

### 4.4. Cell Lines

HUVECs derived from the human umbilical cord vein of healthy donors (*n* = 3) were obtained from the biobank of the Institute for Regenerative Medicine, Lomonosov MSU, collection ID: MSU_HUVEC (https://human.depo.msu.ru). The cells were cultured on gelatin-coated plastic in endothelial growth medium (EGM-2, Lonza) and used for experiments at 2–3 passages.

MCF10A non-tumorigenic epithelial cells (ATCC^®^ CRL-10317™), were cultured in DMEM/F12 medium supplemented with 5% horse serum, GlutaMAX-I (10 μL/mL), EGF (20 ng/mL), hydrocortisone (0.5 μg/mL), cholera toxin (100 ng/mL), insulin (10 μg/mL), NaHCO_3_ (32.5 μg/mL), penicillin (100 U/mL), and streptomycin (100 μg/mL). SKBR3 human breast cancer cells (ATCC^®^ HTB-30™), were cultured in DMEM supplemented with 10% FBS, penicillin (100 U/mL), and streptomycin (100 μg/mL). Some assays were performed in serum-free culture media, and in the presence or absence of EGF.

### 4.5. In Vitro Tube Formation Assay

The effect of plasma exosomes and total blood exosomes from the blood of HFs and BCPs in vitro on capillary-like tubes formation on Matrigel was evaluated. HUVEC were seeded in 24-well plates coated with growth factor reduced Matrigel (BD Bioscience) at a concentration of 35 × 10^3^ cells/ well. Three wells were used for each sample of exosomes. Supplement-free serum-free EBM-2 (Lonza) was utilized as a negative control; EGM-2 (Lonza) with 10% FBS served as a positive control. Plates were placed into CO_2_-incubator at 37 °C and capillary-like structures were assayed in 18 h under the light microscope (Leica, Germany). The total length of tubular structures was counted in 4 random fields of view (objective 10×) with MetaMorph 5.0 software (Universal Imaging).

### 4.6. Cell Migration Assay

Next, 2D migration assays for MCF10A and SKBR3 cells were performed as described previously [35]. To estimate the cell motility, we analyzed the number of migrated cells under each condition (a cell was defined as migrated if it moved a distance longer than its radius) and the total path of moving cells based on tracking the cell migration using the MTrackJ plugin in ImageJ. Two–three fields of view were recorded for each condition in three independent experiments.

### 4.7. Analysis of Proliferation Intensity

The number of mitosis was calculated in the same video of live MCF10A and SKBR3 cells which was obtained from migration analysis to estimate the proliferation intensity.

### 4.8. Immunofluorescent Staining of Cell-Cell Adhesions

For immunofluorescent staining for E-cadherin and β-catenin in cell–cell AJs, cells were plated on 24-well cultural dishes (Corning, USA) with cover glasses under the conditions described above for migration experiments. After the washout of serum and EGF for 5 h the medium was changed to DMEM with or without serum/EGF/exosomes, and the cells were additionally incubated at 37 °C in a humidified atmosphere at 5% CO_2_ for 24 h. For E-cadherin staining, the cells were fixed by 1% PFA/PBS for 2 min and placed in cold methanol for 15 min. For staining to β-catenin cells were fixed in 3.7% PFA for 15 min and then permealyzed with 0.2% Triton X-100 for 2 min. Fixed cells were washed by PBS (3 × 15 min) and incubated for 40 min with primary antibodies and subsequently for 40 min with fluorochrome-conjugated secondary antibodies. As primary antibodies, anti-E-cadherin (BD Biosciences, BD Transduction Laboratories) and anti β-catenin antibodies (Sigma-Aldrich) were used. As secondary antibodies, we used tested class-specific Alexa Fluor 594-conjugated AffiniPure Goat Anti-mouse IgG2a (Jackson Immuno Research), Alexa 488 anti-mouse IgG (Sigma-Aldrich). All the antibodies were diluted according to manual recommendation. Nuclei were stained with DAPI (Sigma-Aldrich).

Cells were analyzed with Zeis Axioplan microscope equipped with Plan Neofluar ×63 oil objective. Images were acquired with the Olimpus DP70 digital camera with the software DP Controller (Olympus, Japan).

### 4.9. Evaluation of microRNA (miRNA) Concentrations

Before the isolation of miRNA, samples of exosomes were thawed and gently mixed. Isolation of miRNA was performed as described previously, precipitated with glycogen and isopropanol, and reconstituted in 30 μL of RNAse-free water [47]. After the addition of denaturation buffer, synthetic cel-miR-39-3p was spiked-in the samples at 5 × 10^7^ copies per isolation.

Each RNA sample was reverse-transcribed to cDNA using the TaqMan^®^ MicroRNA Reverse Transcriptions Kit (PN 4366596, Applied Biosystems, USA). Single-stranded cDNA was synthesized from 2.5 μL of RNA using specific miRNA primers [31,47,48]. Samples without RNA templates were used as negative controls. A total of 4 μL of cDNA was used as a template in a 24 μL PCR reaction. PCR products were amplified using specific primers [31,47,48] and TaqMan Universal PCR Master Mix (PN 4427788, Applied Biosystems, USA) and detected using the CFX 96TM Real-Time System (Bio-Rad, USA). The PCR reactions for each sample were run in duplicate, including blank controls without cDNA. After an initial denaturation at 95 °C for 3 min, the reactions were run for 45 cycles at 95 °C for 15 s and 60 °C for 45 s. The relative expression values of target miRNAs were normalized to miR-16 and were calculated following the 2^-dCt^ method, as described previously [17,31,32]. 

### 4.10. Statistical Analysis

Statistical analysis was performed using the Statistica 6.0 software and the GraphPad PRISM 5 software (GraphPad Software, La Jolla, CA, USA). All the data were expressed as medians with interquartile ranges or as means with standard errors, and the paired data obeying the normal distribution and the homogeneity of variance were compared between two groups with a paired t-test, the unpaired data obeying a normal distribution, and the homogeneity of variance was calculated using an unpaired t-test. Data among multiple groups were compared with a one-way analysis of variance (ANOVA). *p*-values < 0.05 were considered to be statistically significant. The cell motility data represented at least three independent experiments. 

## 5. Conclusions

In summary, the results of the present study indicate that plasma exosomes and total exosomes from the blood of BCPs have differential expression of tumor-associated miRNAs and may induce angiogenesis, EMT, and the motility of breast pseudo-normal and cancer cells, thus consequently supporting tumor dissemination. The multidirectional effect of HF total blood exosomes on tumor dissemination was revealed: they suppress angiogenesis and the total path length of breast pseudo-normal cells and, at the same time, stimulate EMT and the proliferative activity of pseudo-normal MCF10A cells. In addition, the circulating exosomes secreted by normal cells have potency in stimulating the metastasis-promoting properties of breast cancer cells. Taken together, all the total blood exosomes may take part in the regulation of the key steps of tumor progression, including proliferation, EMT, cell migration, and the stimulation of angiogenesis. Our findings may open a new vista for the therapy of breast cancer as well as other cancers.

## Figures and Tables

**Table 1:** Clinical characteristics of the BCPs.

		No (%)
Tumor stage	T1	19 (44)
	T2	21 (49)
	T3	1 (2)
	T4	2 (5)
Nodal status	N0	27 (63)
	N1	9 (21)
	N2	2 (5)
	Nx	5 (11)
	M0	43 (100)
Estrogen receptor status	Positive	30 (70)
	Negative	11 (25)
	Unknown	2 (5)
Progesterone receptor status	Positive	27 (63)
	Negative	14 (32)
	Unknown	2 (5)
HER2 status	Positive	11 (25)
	Negative	30 (70)
	Unknown	2 (5)
Ki-67	<10	15 (35)
	10 ≤ ≤ 20	16 (37)
	>20	10 (23)
	Unknown	2 (5)
Histologic grade	II	41 (95)
	III	2 (5)
Infiltrative ductal carcinoma		43 (100%)
Total patients		43(100%)
